# Supplementary material for: Transcriptome Analysis Reveals Vimentin-Induced Disruption of Cell–Cell Associations Augments Breast Cancer Cell Migration
Source: Cells. 2022 Dec 13;11(24):4035. doi: 10.3390/cells11244035 (PMC9776984; doi:10.3390/cells11244035)
Supplement: Supplementary file 1 [file cells-11-04035-s001.zip › DEGs and enrichment analysis.zip!/DEGs and enrichment analysis/WT_VIMvsVector KEGG/DOWN/WT_VIMvsVector_down.dot.pdf]

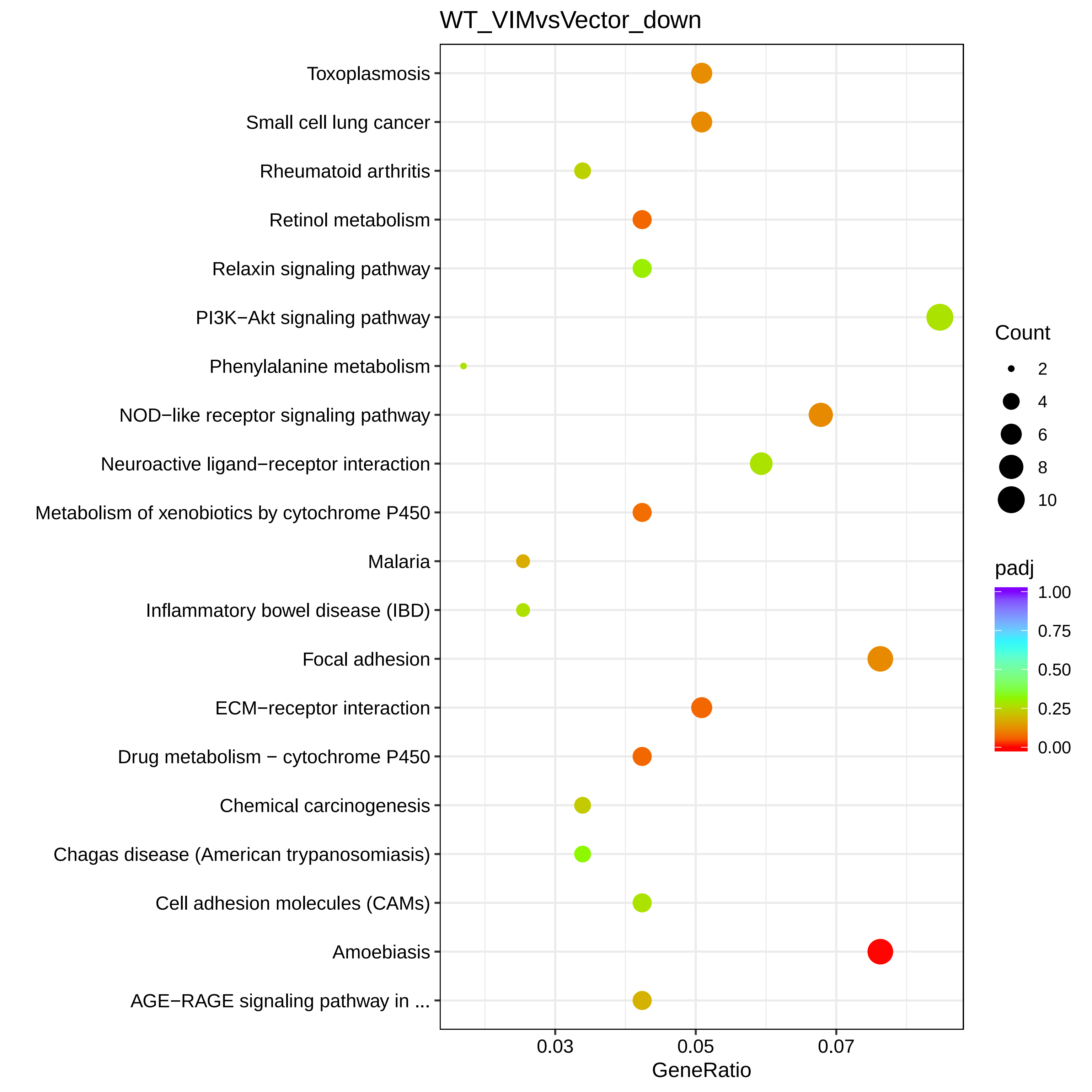

WT_VIMvsVector_down
Toxoplasmosis
Small cell lung cancer
Rheumatoid arthritis
Retinol metabolism
Relaxin signaling pathway
PI3K−Akt signaling pathway
Phenylalanine metabolism
NOD−like receptor signaling pathway
Neuroactive ligand−receptor interaction
Metabolism of xenobiotics by cytochrome P450
Malaria
Inflammatory bowel disease (IBD)
Focal adhesion
ECM−receptor interaction
Drug metabolism − cytochrome P450
Chemical carcinogenesis
Chagas disease (American trypanosomiasis)
Cell adhesion molecules (CAMs)
Amoebiasis
AGE−RAGE signaling pathway in ...
0.03
0.05
0.07
GeneRatio
Count
2
4
6
8
10
padj
1.00
0.75
0.50
0.25
0.00
